# Boy Smokers’ Rationalisations for Engaging in Potentially Fatal Behaviour: In-Depth Interviews in The Netherlands

**DOI:** 10.3390/ijerph15040767

**Published:** 2018-04-16

**Authors:** Michael Schreuders, Nikha T. Krooneman, Bas van den Putte, Anton E. Kunst

**Affiliations:** 1 Department of Public Health, Academic Medical Centre, 1100DD Amsterdam, The Netherlands; n.t.krooneman@vu.nl (N.T.K.); a.kunst@amc.uva.nl (A.E.K.); 2 Faculty of Social and Behavioural Sciences, University of Amsterdam, 1018WV Amsterdam, The Netherlands; S.J.H.M.vandenPutte@uva.nl; 3 Trimbos Institute, Netherlands Institute for Mental Health and Addiction, 3521VS Utrecht, The Netherlands

**Keywords:** adolescent health, adolescent smoking, tobacco, health risk appraisal, cognitive dissonance

## Abstract

Adolescent smokers engage in cognitive rationalisation processes that lower perceptions of personal vulnerability to the health consequences of smoking. There is, however, hardly any evidence that provides in-depth insights on adolescents’ recurring rationalisations. Therefore, we explored how boy smokers deal with the knowledge that they are engaging in potentially fatal behaviour. Interviews were held with 16 boy smokers aged 16 to 17 years old. The qualitative analysis focussed on combining boys’ reasons about why they think they will not experience severe health consequences into coherent rationalisations that recurred among interviewees. Three rationalisations emerged from the analysis. First, boys trivialize the immediate consequences and think these can be compensated for and are outweighed by the benefits of smoking. Second, boys assume that smoking will only take place during adolescence and they will, therefore, recover from the damage inflicted. Third, boys believe that they have control over the amount and frequency of smoking and, thereby, can ensure that they will not experience fatal consequences. Boys’ recurring rationalisations build on their view that they are supposed to have fun and will not become typical adult smokers. Interventions should address these rationalisations in order to increase adolescents’ perceptions of personal vulnerability, and thereby contribute to decreasing adolescent smoking.

## 1. Introduction

In Western countries, most individuals know that smoking is harmful for one’s health. This is, among other things, the result of the many years of health interventions that convey information about the risks connected to smoking. A key argument to convey such information builds on behavioural theories (e.g., health belief model) that posit that improving individuals’ knowledge about smoking contributes to averting or stopping them from smoking [1].

The question of how smokers deal with the knowledge that they are engaging in an unhealthy and even a potentially fatal behaviour (i.e., discrepancy between knowledge and behaviours) has often been addressed in scientific literature. This literature demonstrates that smokers—and even non-smokers who intend to smoke later on [1]—persistently engage in cognitive rationalisation processes [2,3,4] that lead them to decrease their perceptions about the personal health risks of smoking relative to that of other smokers [5,6,7]. Thus, the improvements in smokers’ knowledge about the health risks of smoking have had only a sub-optimal impact because cognitive rationalisation processes decrease smokers’ perceptions of personal vulnerability.

Literature has identified multiple different rationalisations that smokers use to lower their perceptions of personal vulnerability. A key study by Oakes et al. [8] identified four co-occurring categories of such, what they termed self-exempting beliefs: ‘bulletproof beliefs’ when individuals think the risks do not apply to them (e.g., good genetics); ‘sceptic beliefs’ when individuals refute the evidence about the harms of smoking; ‘jungle beliefs’ when individuals argue smoking is just one of the many risks in life; and ‘worth-it beliefs’ when they consider the benefits to outweigh the risks of fatal disease. Helweg-Larsen et al. [9] later identified so-called ‘risk-delay beliefs’ as a fifth category. This belief denotes that smokers emotionally distance themselves from the health consequences as these will only occur in the long-term future.

The scientific argument for explicitly addressing, what has been termed, smokers’ ‘lay knowledge’ [10], ‘self-exempting beliefs’ [8], ‘rationalisations’ [11] and ‘risk denial’ [12] in health interventions is mounting. Peretti-Watel et al. [12] (p. 356) claim that health interventions should not only provide smokers with information about the health risks, but above all prevent ‘them from engaging in many cognitive processes that fuel underestimation of risk’. Fotuhi et al. [11] (p. 57) similarly reason that addressing smokers’ rationalisations will motivate them to quit because ‘if this route of dissonance reduction is cut-off, then the only other remaining way for smokers to reduce their dissonance is to change their behaviour’.

Adolescents’ smoking uptake and persistence is still a major public health concern. A comparison with adults shows that adolescents have a relatively strong tendency to rationalise their smoking behaviour [13]. Literature indicates that adolescents may use a wide range of rationalisations. Adolescents may believe that: smoking can be compensated for by engaging in other healthy behaviours [14]; the health consequences of smoking are negligible; smoking is less bad than other risky behaviours; smoking is worth the risk; they are impervious to the risks [15]; they have control over their smoking behaviour [16,17]; they can smoke for a few years and then quit [18]; and they will quit smoking before it becomes harmful [19,20,21]. There still is, however, little understanding about which rationalisations would come up in adolescents’ minds when thinking about or discussing the health implications of smoking in their ordinary lives (e.g., during tobacco education at schools, discussions with peers). Indeed, studies often presented adolescents with a survey of pre-defined statements that do not necessarily correspond with adolescents’ thinking and also provided little in-depth understanding about how adolescents explain why they adhere strongly to certain rationalisations.

Therefore, we had personal conversations with Dutch boys to explore the recurring rationalisations and understand how they explain why they adhere to these rationalisations. We focussed only on boys, as scholars increasingly plea for more gender-specific knowledge on tobacco use in adolescence. Differences between boys and girls that influence their smoking relate to the many things that make up the everyday experience of being a teenage boy or girl, including “the gendered ‘identity work’ that adolescents undertake to achieve a socially and culturally acceptable image” [22]. We openly talked about these boys’ smoking behaviours and knowledge of the associated health risks, then probed them to explain why they think that they will not suffer from smoking-related health consequences, and thereafter combined these boys’ explanations into coherent rationalisations that recur. The aim was to provide insights that may enable health interventions to become better able to prevent boys from engaging in the cognitive rationalisation processes that decrease their perceptions of personal vulnerability to the health risks of smoking.

## 2. Materials and Methods

The study adopted a qualitative approach because it allowed us to delve deeply into an individual’s personal accounts of what they know about the health risks of smoking and why this knowledge does not make them decide to immediately stop engaging in such unhealthy behaviour.

### 2.1. Brief Contextual Background

Of the Dutch adult population, 26.3% of people smoke; 74.4% of these smokers do so on a daily basis. Statistics on 16-year-old boys show that 11.0% smoked in the month prior to the survey and that 3.6% of all boys smoked on a daily basis in 2015 [23]. Comparable statistics do not exist for 17-year-old boys. This smoking happens despite a national law that prohibits the sale of cigarettes to individuals below 18 years of age.

### 2.2. Sample

We sampled 16 boy smokers who were 16 or 17 years of age and living in the Amsterdam region. Inclusion criterion was that they smoked cigarettes and/or roll-your-own tobacco at least once a week over the last six months. N.T.K. approached smoking boys at their school area directly to make an appointment for participation. N.T.K. also approached adolescents that she did not see smoking to ask them if they are smokers or if they know someone else who smokes. Heterogeneity of interviewees was further realized by sampling boys at seven different secondary schools and balancing the number of interviewees from lower (VMBO: Voorbereidend Middelbaar BeroepsOnderwijs), middle (HAVO: Hoger Algemeen Voortgezet Onderwijs) and higher (VWO: Voorbereidend Wetenschappelijk Onderwijs) educational levels.

The sample size was sufficient to reach data saturation. We started with 12 interviews. Analysis of these 12 interviews showed that the later interviews were hardly providing new codes. We then decided to interview another four boys from different schools and found no new codes.

### 2.3. Design and Procedure

Semi-structured interviews with individual boys were considered most appropriate as this allowed us to elaborate thoroughly how boys rationalise the discrepancy between their views of the health risks of smoking and their smoking behaviour. Also, this ensured that boys gave their answers independent of other boys’ answers. The interview-guide was not informed by rationalisations that were identified in earlier literature, because we chose to probe what the boys said so as to find out what rationalisations boys would think of in their ordinary lives. In order to do so, we were inspired by Helweg-Larsen, Tobias and Cerban [9] and additional information from Helweg-Larsen that we received on request. The said authors interviewed adult smokers about their risk perceptions for the ‘typical smoker’ and themselves, thereby providing the basis for further investigation.

The interviews with individual boys were held by the second interviewer in the Dutch language and lasted approximately 30 min. The interviewer had an informal chat with the boys upon their arrival, after which she told them about the study, personal anonymity and researchers’ confidentiality, explained their rights, and asked for consent and approval for recording the interview. The official interview started with an open question on why they think people (i.e., in general) smoke or do not smoke. The interviewer probed for both ‘positive’ and ‘negative’ consequences of smoking, but specifically emphasized the health consequences. After sufficiently exploring their views of the health consequences, the interviewer steered the interview so that the interviewees started talking about what they think are their personal risks. The interviewer occasionally confronted the interviewees subtly with what they had said in the beginning of the interview so as to make them elaborate on why they think there’s a difference between their own and others’ health risks and continuously probed for further explanation. The interview ended with a question on how they see their own tobacco use in the future.

After the interview, participants were asked to answer a brief survey entailing questions on personal characteristics, the number of cigarettes or packs of cigarettes smoked per week, smoking self-categorization (i.e., do you consider yourself a light, average or heavy smoker?) and statements about knowledge of the health risks of smoking (e.g., do you think that smoking increases the risk of cardiovascular diseases?).

### 2.4. Analysis

The voice recordings were transcribed verbatim for analysis. The analysis focused on finding recurring rationalisations on how boy smokers explained the discrepancy between their knowledge about the harms of smoking and the fact that they are smoking. The first step entailed the coding of any reason that they put forward explaining why they think they will or will not experience the health consequences of smoking. In the second stage, the reasons they put forward were combined into three coherent rationalisations that recurred across the interviewees.

All transcripts were read and coded individually by N.T.K. and M.S. Bi-weekly discussions with the said authors and A.E.K. were employed to reflect on the coding and subsequent emergence of recurring rationalisations. Differences in coding were discussed and solved. Agreement between all authors led to the three rationalisations presented in the Results section. All quotes used in the Results section were translated into English as accurately as possible and kept in context.

The datasets used and/or analysed during the current study are available from the corresponding author on reasonable request.

The AMC Medical Ethics Review Committee reviewed the study proposal and concluded that the Medical Research Involving Human Subject act (WMO) does not apply to this study and that an official approval by this committee was not required (letter W16_068). All adolescents provided written consent for participation.

## 3. Results

### 3.1. Participants’ Characteristics

All participants were 16- or 17-year-old boys. Five boys were at a lower, six a middle and five a higher educational level. The number of cigarettes smoked per week ranged from between 7 and 95. Most boys considered themselves to be light or average smokers. The only person who considered himself to be a heavy smoker smoked 35 cigarettes a week, but underpinned his view by saying that he is very motivated to stop and thinks every cigarette he smokes is one too many. All boys had at least two of the three knowledge statements correct. Table 1 presents an overview of participants’ characteristics.

### 3.2. Recurring Rationalisations

The analysis found three recurring rationalisations that explain how boy smokers cognitively deal with the discrepancy between their knowledge about the harmfulness of smoking and the fact that they are smoking. This section elaborates on these separately.

*Rationalisation 1: Immediate consequences are trivialized*, *can be compensated for*, *and are outweighed by the benefits of smoking*.

Boys noticed that smoking immediately impacts their health. They often recalled negative consequences for their physical endurance, teeth, skin, sense of smell and taste. Outbursts of smoking were often accompanied with, what they call, ‘a smokers’ cough’ the day(s) after.

‘Normally (before I started smoking) I could play a full match (soccer), but now only three quarters of a match. Or half a match, if I’ve really smoked a lot.’

(#8)

While recognizing such immediate consequences of smoking, most boys were not bothered too much by these consequences as they constantly trivialize its impact on their lives.

‘Well, if I smoke just before I have to cycle, then I notice it, I have to cycle slower because otherwise I sit on my bike with a hoarse gasp … you know … that is not nice. So mostly I do not smoke before cycling. Hahaha (laugh).’

(#9)

‘I do not notice it too much. Yes, maybe during sports. But I do not do sport at a high level, so these are not consequences that make me think …’

(#12)

‘I am a dancer and the smoking influences my condition and the power in my movements. It all has become more difficult, but I don’t really notice it.’

(#16)

The finding that boys are not bothered too much by the immediate consequences also stems from their view that these can be compensated for by executing other healthy behaviours (e.g., doing sport, eating healthily, increasing the brushing of teeth etc.).

‘You can just keep up your teeth (with proper dental care) when smoking, I am the living proof.’

(#5)

Smoking, moreover, brings about benefits that they value more than said direct consequences.

‘(The impact of smoking on) my condition and taste and so on, that is really … but on the other side it (smoking) is a very sociable thing to do, it tastes good and I really like to do it.’

(#1)

There were, in fact, only very few boys who argued that the immediate health consequences make them want to stop smoking. This intention to stop due to the direct consequences particularly applied to those who feel seriously hampered in their physical endurance.

‘I play sports a lot and you just notice that after going out in the weekend and drinking and smoking a lot, that your endurance is bad (…) This is a big reason for me, and the money, to stop smoking.’

(#11)

*Rationalisation 2: Smoking only takes place during adolescence*, *so the body will recover from the inflicted damage*.

Boys reasoned that the damage done by smoking is a function of the number of cigarettes that someone smokes over the course of life. Most boys assumed that the number of cigarettes they (will) smoke are too few to increase the chances of developing a fatal disease considerably.

‘I just don’t think my chances of getting it (lung cancer) really increase (in comparison to non-smokers) with the amount that I smoke.’

(#6)

‘I think my smoking is fine … just not smoke too much.’

(#10)

Boys, indeed, primarily associated risks of such fatal consequences with individuals who smoke ‘many cigarettes’ for ‘a long period of time’—the meaning of which varied across individuals. They assumed that individuals who have been smoking for a short period will largely—apart from ‘some (often undefined) permanent damage’—recover from the damage that has been inflicted.

‘I have just been smoking for four years while there are also people that have been smoking for their entire life. These individuals will get lung cancer, and I think that if I stop now I will not experience consequences anymore (…) on the internet you also see that your health slowly gets better, and that it is good again in 20 years or so’

(#1)

‘Yes, (severe consequences) particularly (occur) if you keep on smoking, I think that if you quit, it (my health) will slowly get better (…) my grandma, her lungs were totally black, but they got clean again.’

(#11)

‘You will always experience some effects (health consequences from smoking), but I think that you can minimize the impact of these effects so that you do not really notice it. I will probably have a lower lung capacity or higher blood pressure, but I think this will become better if you start living healthy.’

(#12)

Most boys said that they will stop smoking in the following months or years. The conviction that they will stop is fundamentally built on their view that smoking is part of adolescence. They typified adolescence as a phase in life where they can still get away with deciding to favour experiencing ‘joy’ over the ‘wise decision’ not to smoke.

‘By then (when planning to get children) it will not have any value anymore. Now, I am still young and can just behave the way I want.’

(#7)

‘I know it is bad for you. But it is just … it is sociable and I mean … why not just enjoy something when you are young?’

(#9)

Some boys even said they will adopt a similar stance on smoking if their future children start smoking in adolescence.

‘Yes (my children will be allowed to smoke), because it would be hypocritical to forbid them (…) they can experiment with it, but then I will tell them that I had fun smoking for a couple of years and then quit. So (I will tell them) they should try that.’

(#15)

Boys, in contrast, talked about adulthood as a phase of life in which they have to become more serious and responsible, and therefore it seemed self-evident to them that they will quit smoking. They characterised adulthood by decreases in the amount of social events, the necessity to work on a professional career, and also the idea of being a responsible (i.e., non-smoking) dad for their future children.

‘You know … now everything is still exciting, I am not 18 years of age and everybody around me smokes at parties (…) so it is just part of it (life at this moment). At a certain point in time you stop going to parties every weekend and your life becomes more serious. I am sure that I will not keep on smoking.’

(#5)

‘I think (I will quit smoking) after my studies. When I start a responsible life.’

(#13)

‘Then I will leave secondary school. All my friends will also be gone. Then I won’t see anyone smoking and get new friends (…) that is a new beginning, just (make sure that I do) not start (smoking) again.’

(#15)

Similarly, some boys found it irresponsible and immature to see adults smoking. This was particularly the case for those with no smoking in the family.

‘I do not see myself smoking as an adult. If you smoke as an adult I think that is immature. If I am adult, I will act like an adult (…) If my parents would smoke it would not make any sense … they’re a role model to someone.’

(#5)

*Rationalisation 3: Health consequences can be prevented as long as there is control over the amount and frequency of smoking*.

Boys argued they can prevent the health consequences of smoking as long as they remain in control over the amount and frequency of smoking. Specifically, control over smoking gave them the feeling that they have an influence over the occurrence of health consequences. Illustrative of this reasoning, one interviewee mentioned that he would like to have a status report of his current health situation so as to be able to act on it (i.e., reduce or quit smoking).

‘I am not really worried (about the health risks), but I would like to know the current situation (of the damage done by his current level of smoking), so that I know how it (the damage) will progress or just remain the same (as now) if I continue smoking the way I am doing now.’

(#5)

Boys—even those who considered themselves to be addicted—were in a continuous search for control over their smoking behaviour. This search manifested itself in several tactics: limiting the amount they smoke each day, minimising the buying of cigarette packs themselves, refraining from smoking for certain periods of time, or abstaining from smoking when they are with their families. Successful experiences with such tactics were persistently put forward as evidence underpinning their control.

‘I know that I will eventually decrease the amount of smoking. I also have periods where I do not smoke for three months, so I know that I can.’

(#3)

‘I managed not to buy cigarettes for about a year, just for the idea that I would not get addicted if I wouldn’t have them nearby.’

(#8)

These successful experiences were also put forward to underpin why they believe they will succeed in quitting smoking later in life—even if, in fact, the situation was a failed attempt to quit.

Interviewee: ‘Yes, I am a little afraid (that I will be too addicted to quit) (…) but the largest part is mental, and if you think ‘I don’t want this anymore’… I also quit cold-turkey one time before and stopped smoking for three months, until my exams started.’

Interviewer: ‘So if you really want to, you will succeed?’

Interviewee: ‘Yes, I will.’

(#11)

‘I have often quit for half a year and this gives me the feeling of knowing that I can stop at any moment.’

(#13)

In contrast, social situations were often referred to as instances where smokers had less control over their smoking. This, however, did not concern them too much as it was different from how they act ‘normally’ (e.g., just in holidays or weekend).

‘In holidays you smoke a pack a day and then you think ‘shit … what am I doing?’ (…) I need to start smoking less for a while.’

(#7)

‘I think I am an average smoker, but when I go out I sometimes smoke an entire pack. Certainly, in combination with alcohol, then it is three times as nice so then you smoke even more.’

(#9)

‘In the weekends I sometimes buy a pack before going out for a drink because I will share it and smoke not all of it myself.’

(#11)

Referring to the difficulties of having control in social situations, one boy explained how he tries to manipulate his friends—instead of simply saying ‘no’—to limit the number of cigarettes (i.e., tactic to underpin control) that he smokes.

‘When I am at school with friends and they say ‘we’re going for a smoke’ and I do not feel like it, I say ‘let’s just wait for a bit’. This waiting can easily last an hour, leaving no time for smoking because of a new school hour that is starting. This is how I secretly try to manipulate.’

(#15)

## 4. Discussion

Literature indicates that adolescent smokers engage in cognitive rationalisation processes to deal with the knowledge that they are engaging in a potentially fatal behaviour. We explored how boy smokers rationalise the discrepancy between their knowledge about the health risks of smoking and the fact that they are smoking. The results showed that these boys are, indeed, largely aware of the negative health consequences, but persistently put forward three recurring rationalisations.

The first rationalisation was that boys trivialize the immediate consequences and think these can be compensated for and are outweighed by the benefits of smoking. Boys referred to negative consequences for their physical endurance, teeth, skin, sense of smell and taste, but these did not make them want to stop smoking during adolescence. Only boys who (plan to) undertake sports at a serious level considered the immediate consequences hampering them to such an extent that they wanted to quit smoking quickly.

The second rationalisation was that boys assume they will smoke only during their adolescence and so afterwards they will largely recover from the damage that has been inflicted. Boys connected the fatal consequences only to individuals who smoke many cigarettes for a long period of time. They were confident they will not do so because smoking is part of the many social events that are inherent to adolescence, but is irresponsible and immature when one becomes an adult.

The third rationalisation was that boys believe themselves to be in control over their amount and frequency of smoking and so can avoid suffering fatal health consequences. They employed several tactics and put forward successful experiences with those tactics as evidence to nurture the belief of control. Social situations were, however, often referred to as instances where they had less control, but this did not bother them too much as it was considered to be different from how they act normally (e.g., just in the holidays or at weekends).

## 5. Limitations

We focussed on smoking boys’ cognitive attempts to deal with the health risks of smoking. This focus on health risks was a deliberate decision, but in the transcripts we found some referrals to the social benefits and costs of smoking (e.g., ‘I want to stop because I fell in love with a girl who doesn’t like it when I smoke’ #8). This corresponds with research showing the importance of social outcome expectancies on adolescents’ smoking status [24]. Future studies that focus on social benefits and costs as well as health risks would produce a more comprehensive representation of adolescents’ cognitive dealings with their smoking behaviour.

The study did not include boys who never smoked but do have the intention of smoking. Including them would have been interesting, as research shows that those with smoking intentions (i.e., susceptible non-smokers) also have lower perceptions of personal risk [1]. Such evidence would have allowed us to understand how rationalisations develop from the susceptibility to the smoking stage and see whether different health interventions are needed to prevent adolescents in different stages of smoking uptake from engaging in cognitive rationalisation processes.

Lastly, we should be cautious in generalizing these results about 16 Dutch boys from the Amsterdam region to other adolescents and contexts. Next to sample size and locality, another reason to be cautious is that we did not explore whether rationalisations are different among individuals with different characteristics. Characteristics that could influence the rationalisations are adolescents’ gender, length of smoking, ethnicity, self-perceived addiction, amount of friendship ties, and dual use of tobacco products.

### 5.1. Comparison with Adults

This study identified three recurring rationalisations that boys use to decrease their perceptions of personal risk. These recurring rationalisations logically fit with boys’ brief smoking histories, stage of current smoking, and developmental phase. Relative to most adult smokers, 16- and 17-year-old boy smokers have smoked for a shorter period of time, smoke on average fewer cigarettes a day, more often smoke in social situations, have fewer responsibilities, and are still in the midst of their social and cognitive development [25]. Therefore, it is no surprise that boys’ recurring rationalisations fundamentally build on the view that they are supposed to have fun and will not become the *typical smoker* who—in their understanding—risks dying from its connected diseases. This view underlying boys’ rationalisations may also occur more generally among adolescents in The Netherlands and similar countries.

We suggest that boys’ view that they are supposed to have fun and will not become a typical smoker manifests itself in putting more weight on different rationalisations than adults. Noteworthy is boys’ strong emphasis on worth-it and bulletproof beliefs in combination with the insignificance of sceptic and jungle beliefs. This combination denotes that boys feel no need to deny (sceptic) or downplay (jungle) the risks of smoking because they strongly believe that their current acts and future decisions allow them temporarily to enjoy the short-term benefits (worth-it) and safeguard them from the long-term harm of smoking (bulletproof). In contrast, adults often put forward counter-evidence based on personal experiences (sceptic, e.g., my grandfather grew old while smoking all his life) and present smoking as one of the many possible risk behaviours (jungle, e.g., eating unhealthily) [10]. This difference fits with research showing that adults believe less strongly that they can quit smoking whenever they want [18] and consequently need to deal cognitively with the risks that are associated with their likely continuation of smoking.

### 5.2. Reflections

The main contribution of our study is that many existing but dispersed findings on the ways boys’ cognitively deal with the health risks of smoking are combined into three coherent rationalisations that recur among individuals. These rationalisations show not only what boys believe, but also explain why they believe what they believe and how they use or misuse everyday events to maintain their beliefs. We will now describe how the three recurring rationalisations relate to findings in earlier studies.

The first rationalisation indicates that boys do not deny that smoking causes immediate health consequences, but simply considered these not to be sufficiently bothersome to stop smoking during adolescence. This indifference to immediate health consequences (i.e., the ‘costs’ of smoking, leading to worth-it beliefs) corresponds with prior findings showing that adolescent smokers think the immediate health effects are ‘insignificant, manageable and minor’ [15] (p. 578), attach less value to health than non-smokers [26], and adhere relatively strongly (vs. adults) to rationalisations that trivialize the value of health [13]. Boys’ indifference counters some scholars’ pleas to shift the content of health warnings (e.g., on cigarette packs) from the long-term consequences to the immediate consequences of smoking because these are assumed to be more relevant to adolescents [13]. In our study, this relevance only seemed to apply to boys who feel their smoking seriously hampers them in executing sports.

The second rationalisation shows that boys assume that they will stop smoking before adulthood to avert the occurrence of fatal diseases. Adolescents in other studies similarly argue about stopping smoking before becoming adults because this is before severe damage has been inflicted [19]. This corresponds with a recent longitudinal study that shows adolescents are most strongly biased in their perceptions of becoming addicted, relative to other biases [27]. Boys, moreover, explicitly framed smoking as an *adolescent behaviour* as they consider it immature and irresponsible to perform unhealthy acts like smoking in adulthood. This framing of smoking contradicts an earlier qualitative study arguing that children and adolescents associate smoking with adulthood [28]. Our finding that such framing does not apply to Dutch boys corresponds with literature that shows only 11.0% of 13- to 17-year-old Dutch adolescents considered smoking a marker of adulthood in 2009, relative to 46.3% and 68.2% for drinking alcohol and having sexual intercourse, respectively [29].

The third rationalisation denotes that boys—even those who feel addicted—consider themselves to be in control over the amount and frequency of their smoking behaviour and thereby are able to avert fatal consequences. Such naïve feelings of control are part of adolescents’ developmental phase (i.e., egocentric thinking) and nurtures their view of personal invincibility [30]. The theme of control has also been found in prior literature on adolescent smoking. In one study, smoking girls described ‘how they could control their cigarettes, rather than have their cigarettes control them’ [31] (p. 293). Baille et al. [16] (p. 105), accordingly, concluded that ‘youth go to great lengths to monitor their cigarette use carefully’ in order to feel in control. Like in our study, other literature shows that adolescents’ tactics to self-confirm such control include: smoking not too often, not too much, or only on social occasions [16,32,33] and not buying packs of cigarettes themselves [17]. This control gives adolescents the feeling they can change their behaviour in case they would notice that smoking becomes a problem [17] and dangerous to their health [16]. The fear of losing control has, therefore, been reported as a motivator for college students to quit smoking [34] and our study suggests that the same may apply to adolescents.

### 5.3. Key Areas for Future Research

Boy smokers use three recurring rationalisations to decrease their perceptions of personal vulnerability for the health risks of smoking. A main challenge of health interventions is to avert adolescents from engaging in these rationalisations. We now briefly outline three key areas for future scientific inquiry to support the design of such health interventions.

First, future research should explore how health interventions can effectively address adolescents’ rationalisations in order to improve their perceptions of personal vulnerability. Most scholars argue that cutting off rationalisations will likely decrease smoking behaviour, because the only remaining way to reduce cognitive dissonance is to change the behaviour [11]. Some scholars, however, also mention the possibility that rationalising is a robust cognitive skill, so that solving one rationalisation simply leads to the onset of another and, therefore, has no impact on behaviour [4]. In line with the former position, health interventions that elicit cognitive dissonance have shown promising short-term impacts on attitudes, intentions and health behaviours [35].

However, there remains much to be learned on how health interventions can best address adolescents’ rationalisations in order to have a long-term impact on their smoking behaviour—and not merely initiate the development of new rationalisations. Future studies should specifically focus on which smoking stage is most effective for tackling rationalisations (e.g., those who do not smoke, recent smokers, those who want to quit); what type of interventions are most capable of tackling rationalisations (e.g., motivational interviewing, classroom sessions); and in what setting these interventions are best delivered (e.g., school setting, general practitioner). Second, future research should develop more understanding on how to deal with the adverse impacts of health interventions that aim at motivating smokers to quit. This is important, as our results show that boys misuse correct epidemiological information (e.g., smokers who quit smoking will largely recover from the damage) to underpin their rationalisation that it can do no long-term harm if they smoke for a few years during adolescence. Similar misuse of epidemiological facts was reported for adults [12].

Third, future research should develop insights that allow health interventions to better convince adolescents that smoking is addictive. Current health interventions often stress the addictive nature of tobacco smoking (e.g., warning on cigarette packs), yet our results showed that boys still have naïve views about the chance that they may become addicted. We suggest that explicitly countering adolescents’ views that they have control over and will quit smoking could convince them about their personal vulnerability to becoming addicted.

## 6. Conclusions

Boy smokers’ recurring rationalisations fundamentally build on their view that they are supposed to have fun and will not become a typical adult smoker who is likely to suffer from fatal diseases. Addressing these rationalisations might improve boys’ perceptions of their personal vulnerability to the health risks of smoking, but much is still to be learned on how this can be done most adequately.

## Figures and Tables

**Table 1:** Overview of participants’ characteristics.

Code	Educational Level	Self-Classification	Cigarettes per Week	Knowledge Correct
1	Higher	A	70	3/3
2	Middle	L	14	3/3
3	Middle	A	28	3/3
4	Higher	L–A	49	2/3
5	Middle	L	7	2/3
6	Middle	A	19	3/3
7	Higher	L–A	38	3/3
8	Middle	A	27	2/3
9	Lower	A	38	3/3
10	Lower	A	29	2/3
11	Lower	H	35	3/3
12	Higher	A	95	3/3
13	Higher	A	29	3/3
14	Middle	A–H	95	3/3
15	Lower	L	19	3/3
16	Lower	L–A	57	3/3

Self-classification codes: L = light, A = average, H = heavy.
